# Why and when was lactase persistence selected for? Insights from Central Asian herders and ancient DNA

**DOI:** 10.1371/journal.pbio.3000742

**Published:** 2020-06-08

**Authors:** Laure Segurel, Perle Guarino-Vignon, Nina Marchi, Sophie Lafosse, Romain Laurent, Céline Bon, Alexandre Fabre, Tatyana Hegay, Evelyne Heyer

**Affiliations:** 1 Eco-anthropologie, Muséum national d’Histoire naturelle, CNRS, Université de Paris, Paris, France; 2 Aix Marseille University, INSERM, MMG, Marseille, France; 3 APHM, Hôpital de la Timone Enfant, Service de Pédiatrie Multidisciplinaire, Marseille, France; 4 Institute of Immunology and Human Genomics, Academy of Sciences of Uzbekistan, Tashkent, Uzbekistan

## Abstract

The genetic adaptation of humans to the consumption of milk from dairying animals is one of the most emblematic cases of recent human evolution. While the phenotypic change under selection, lactase persistence (LP), is known, the evolutionary advantage conferred to persistent individuals remains obscure. One informative but underappreciated observation is that not all populations whose ancestors had access to milk genetically adapted to become lactase persistent. Indeed, Central Asian herders are mostly lactase nonpersistent, despite their significant dietary reliance on dairy products. Investigating the temporal dynamic of the −13.910:C>T Eurasian mutation associated with LP, we found that, after its emergence in Ukraine 5,960 before present (BP), the T allele spread between 4,000 BP and 3,500 BP throughout Eurasia, from Spain to Kazakhstan. The timing and geographical progression of the mutation coincides well with the migration of steppe populations across and outside of Europe. After 3,000 BP, the mutation strongly increased in frequency in Europe, but not in Asia. We propose that Central Asian herders have adapted to milk consumption culturally, by fermentation, and/or by colonic adaptation, rather than genetically. Given the possibility of a nongenetic adaptation to avoid intestinal symptoms when consuming dairy products, the puzzle then becomes this: why has LP been selected for at all?

## Abstract 2

The genetic adaptation of humans to the consumption of animal milk is a textbook example of gene-culture coevolution. Taking advantage of the accumulated ancient DNA data, this Unsolved Mystery article explores where and when lactase persistence emerged. The mutation rapidly spread throughout Eurasia between 4000 and 3000 BP, but if Asian herders managed to culturally adapt to milk consumption, then why have Europeans genetically adapted at all?

## Lactase persistence, a clear-cut case of human adaptation?

One of the genetically best known examples of genetic adaptation in humans is the appearance of lactase persistence (LP) in some populations, a phenotype characterized by the maintenance of the lactase gene expression throughout adulthood. This allows individuals to digest the lactose found in milk not just during their childhood but throughout their lives. LP has reached high frequencies in various European, African, and Arab populations whose ancestors have domesticated animals and used their milk since the Neolithic revolution, about 5,000 to 10,000 years ago [1]. On the contrary, LP frequency is close to null in areas where human populations did not domesticate animals, or domesticated ones for which milk can’t be used (as throughout the Americas, in Oceania, and in East and South-East Asia). This led to the cultural-historical hypothesis [2,3], which suggests that populations having access to milk selected for LP due to the nutritional gain to obtain glucose out of lactose, while nonpersistent individuals drinking milk suffered from abdominal cramps and potentially life-threatening diarrhea because of the presence of lactose in their colon [4]. Despite being a textbook example of gene-culture co-evolution, the evolutionary advantage conferred by LP has been the object of a long-standing and lively debate [1,5–11], which is not resolved to date. Is this really about caloric intake or rather calcium deficiency [5,12], hydration [13,14], or infectious diseases [12]?

## Central Asia, the exception that proves the rule?

One intriguing observation, which has been largely underappreciated until now, is that in Central Asia (in its broad definition, i.e., including not only the former Soviet Central Asian republics but also Mongolia, Western China, and southeastern Russia), a land where pastoral populations heavily rely on dairy products, the frequency of LP is low, suggesting that these populations have not adapted genetically to digest lactose. Indeed, Mongol and Kazakh herders have LP phenotypic frequencies of 12% to 30% [15,16], despite 35% of the dietary energetic intake in summer deriving from dairy products in contemporary Mongols [17] and despite the evidence of mare’s milk consumption by pastoralist populations associated with the Botai culture in North Kazakhstan as early as 5,500 before present (BP) [18]. As a result, the correlation between LP frequencies and levels of pastoralism is significant at a worldwide scale [8] but not in Eurasia [11].

**Fig 1:**
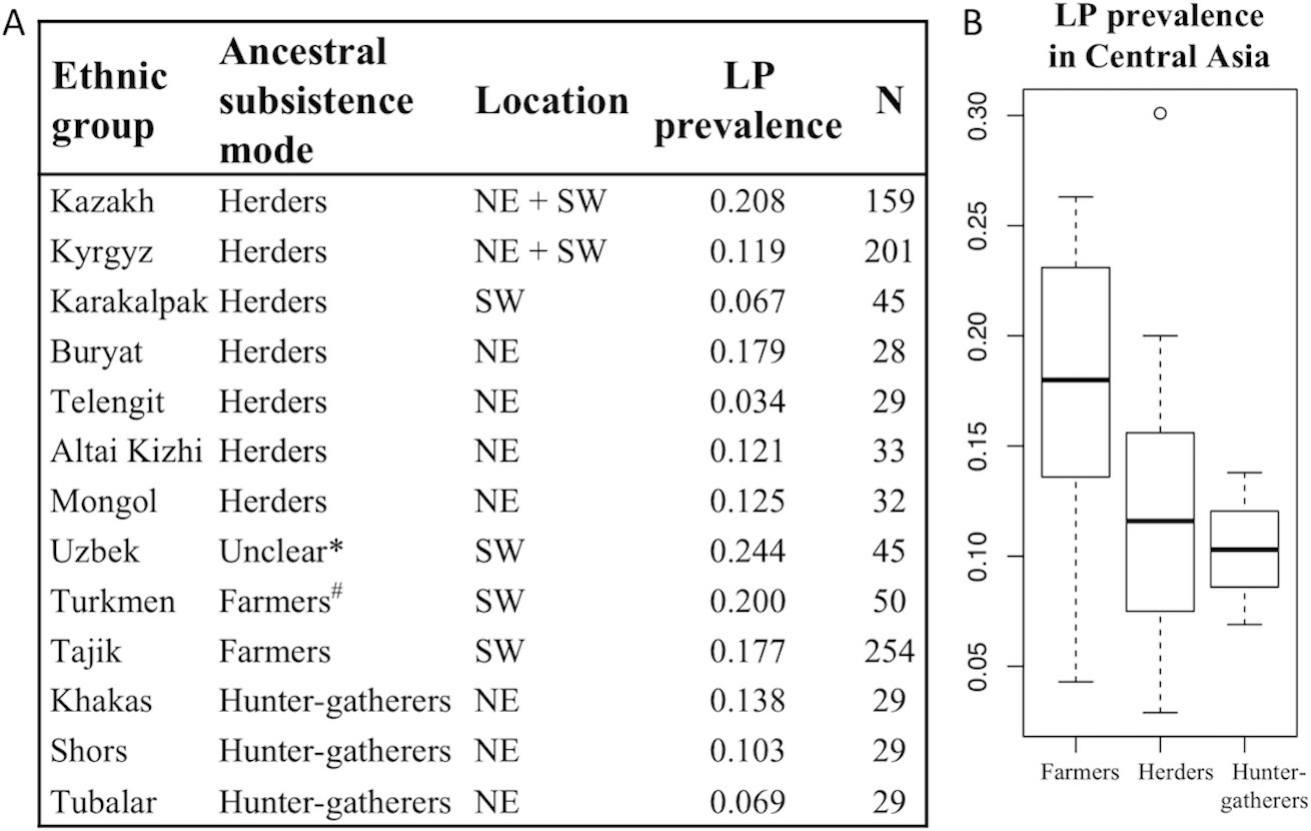
Frequency of LP in various ethnic groups from Central Asia inferred from genotyping data (−13.910:C>T). (A) Frequency per ethnic group. (B) Average frequency per subsistence mode. *It is unclear whether Uzbeks have been mostly herders or famers in the past. They were not included when grouping populations by subsistence modes. ^#^Turkmen are currently herders but were shown to be genetically Indo-Iranian [60] and were suggested to be former farmers who later experienced a culturally mediated change of subsistence mode. “N” is the number of individuals genotyped. LP, lactase persistence.

In order to clarify the situation in Central Asia, we obtained data on the prevalence of LP in modern populations with contrasted ancestral lifestyles (herders, farmers, hunter-gatherers) by genotyping a total of 963 individuals from 30 populations corresponding to 13 ethnic groups (Fig 1A, see Methods). Grouping populations by subsistence mode, we found that herders have an average LP frequency of 12.2% (*N* = 527), farmers of 17.5% (*N* = 304) and hunter-gatherers of 10.3% (*N* = 87) (Fig 1B). Farmers, who rely less on pastoralism than herders, thus present an unexpectedly higher prevalence than herders (*t* test per ethnic group, *p* = 0.035; per population, *p* = 0.081). Within herders, we found a large variability between ethnic groups (from 3% to 21%, see Fig 1A), which is also found between populations within ethnic groups (e.g., within Kyrgyz from 4% to 20%). This variability does not seem to be explained by obvious cultural differences or by a particular geographic pattern. Northeastern and southwestern herder populations indeed do not have significantly different values (10.6% and 13.1%, respectively; *t* test per population, *p* = 0.473). In conclusion, modern Central Asian populations present overall low frequencies of LP, with the highest frequencies observed in farmers. How can we explain this pattern?

## Temporal dynamic of LP in Eurasia

A characteristic of Central Asia is to have been dominated by large-scale populations’ movements and admixture events, which might have impeded the genetic adaptations of these groups. The Botai populations from Kazakhstan, the first to have drunk mare’s milk, indeed seem to have been mostly replaced by the so-called steppe Bronze Age populations (5,000–3,000 BP) that expanded eastward [19–21]. In parallel, a horse-based pastoralist lifestyle has been adopted de novo by some East Asian hunter-gatherers 3,500 BP [17,22], which then expanded westward [23]. It is thus possible that the low LP prevalence observed today in Central Asia is due to the partial replacement of local LP populations by eastern neo-pastoralists (that likely derived from lactase nonpersistent hunter-gatherers).

To investigate this possibility and more generally explore the dynamic of the −13.910*T allele through time in Eurasia, we took advantage of the rapid accumulation of ancient DNA data to directly assess the frequency of the −13.910*T allele in human remains. Following the spatial and temporal evolution of LP is also interesting in light of the debate on where the −13.910:C>T mutation originated. Indeed, the mutation was first suggested to have emerged in farmers from the Neolithic Linearbandkeramik (LBK) culture because it was estimated to have been selected for 7,500 years ago in Central Europe [9] and because the haplotype currently associated with it was found in a 7,000-year-old early European farmer from Stuttgart [24]. However, the same haplotype is also currently found without the T allele [25], therefore imputation might not be reliable. Furthermore, ancient DNA studies found that the LP mutation was absent or very rare in Europe until the end of the Bronze Age [26–29] and appeared first in individuals with steppe ancestry [19,20]. Thus, it was proposed that the mutation originated in Yamnaya-associated populations and arrived later in Europe by migration of these steppe herders.

**Fig 2:**
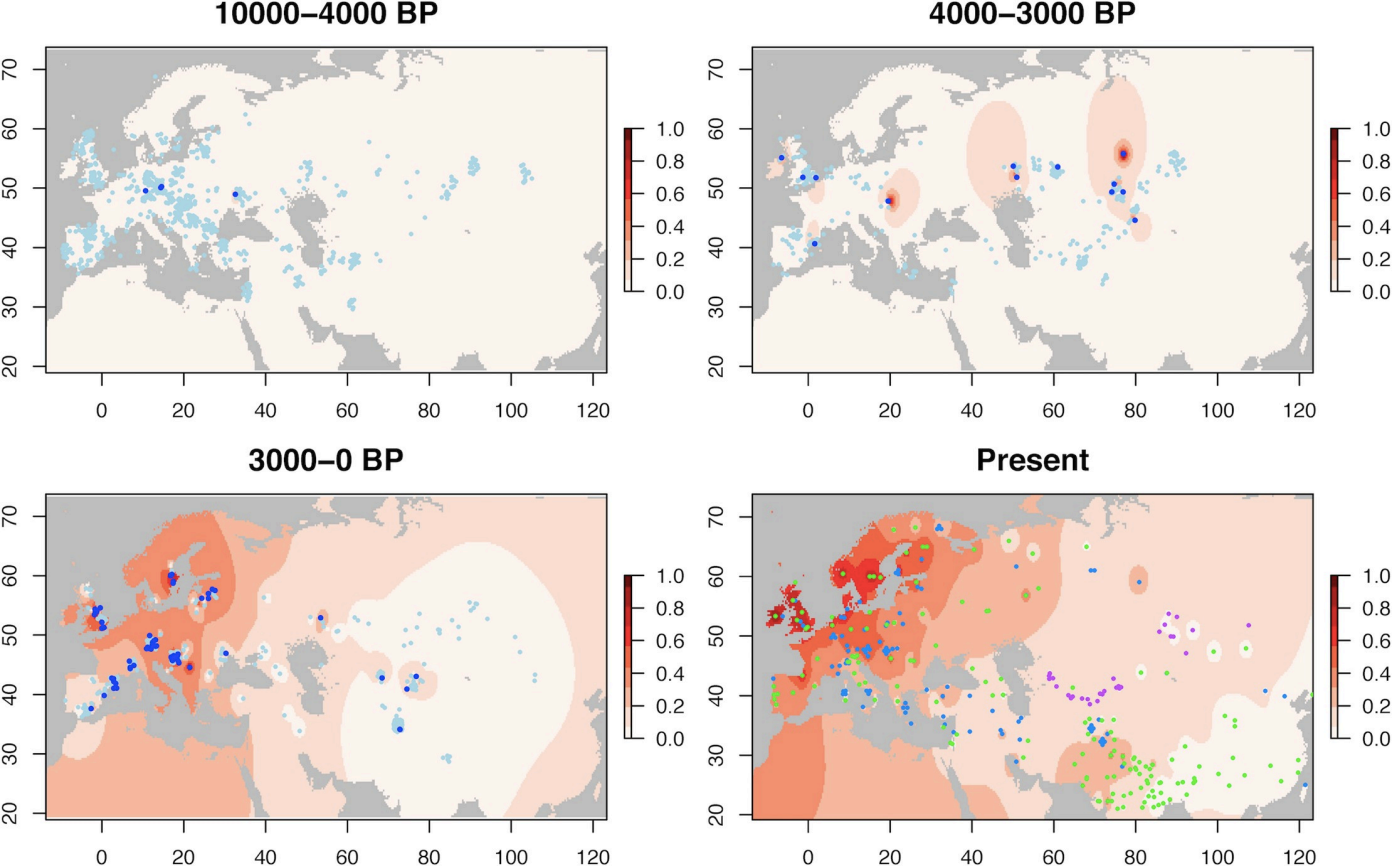
Temporal dynamic of the −13.910*T allele frequency in Eurasia. The color scale represents the extrapolated T allele frequency. For the 3 ancient maps, light/dark blue points represent individuals for which one randomly chosen read carries the C/T allele. For the modern map, purple points correspond to genetic data from our laboratory [11,16,57]; green points represent genetic data based on [61,62] (including only populations for which −13.910:C>T is the only known mutation associated with LP, i.e., all of Eurasia excluding the Arabian Peninsula), and blue points represent inferences of the T allele frequency from direct phenotypic measurements (from the GLAD database [63], as well as from [55], merging what the authors define as LP and lactase-intermediate individuals). These maps were obtained by modifying an R script originally written and shared by I. Mathieson. BP, before present; GLAD, Global Lactase persistence Association Database; LP, lactase persistence.

To address these questions, we extracted the genetic information at the −13.910:C>T mutation (rs4988235) in 1,434 ancient Eurasian individuals ranging from 10,000 BP to present day (Fig 2, S1 Fig and S1 Table, see Methods). The earlier reliable LP individual is a Ukrainian Eneolithic individual dated to 5,960 BP, as previously noted [30], which presents a mixture of Anatolian farmers and steppe ancestry [30]. During the 5,000–4,000 BP period, we see 3 additional LP individuals in Europe (frequency of the T allele of 3/259 = 1.2%) but no LP individuals in Central Asia (though 0/64 is not significantly different from 3/259, proportion test: *p* > 0.89). We don’t see any LP individual among those directly affiliated with Yamnaya-associated cultures, but there are not many of them (48 individuals) compared to non-Yamnaya Europeans (and 0/48 is not significantly different from 3/252, proportion test: *p* = 1).

After its appearance in Central Europe, the T allele then emerges during the 4,000–3,000 BP period concomitantly in a large geographical area in Eurasia ranging from Spain to eastern Kazakhstan (overall T allele frequency of 13/280 = 4.6%, with no significant difference between Europe and Central Asia, *p* = 1) (Fig 2 and S1 Fig). Regarding Asia, the earlier reliable LP individual is one from Kazakhstan dated to 3,713 BP. Then, there is a change in dynamic around the Iron Age, with the T allele rising up in frequency between 3,000 BP and present day in Europe (61/198 = 30.8%) while staying low in Central Asia (4/72 = 5.6%) (proportion test: *p* = 3.6 × 10^−5^). In parallel, the T allele emerges in Pakistan, with the first reliable LP individual dated to 2,250 BP.

In conclusion, in Central Asia, what clearly appears is that the mutation was already present at the end of the Bronze Age and has remained around 5% in frequency since the Iron Age. Thus, if the mutation was under strong selective pressure, it should have had enough time to reach high frequencies today. Indeed, considering a coefficient selection of 4% starting 3,000 years ago on a mutation at an initial frequency of 5%, the mutation should have reached a frequency of 51% (meaning a phenotypic prevalence of LP of 76%) in modern populations. It thus suggests that LP was not strongly under selective pressure in Central Asia, contrarily to the situation in Europe, Africa, and the Arabian Peninsula.

## Cultural adaptation by milk fermentation?

In what ways are Central Asian pastoralists different from other worldwide pastoralists regarding their consumption of milk? First, Central Asia is the first place of horse domestication [18] and the only place where mare’s milk is traditionally drunk, even though there is a high diversity of dairying animal species in the region (horse, cow, yak, sheep, goat, and/or camel). What is interesting about mare’s milk is that it is one of the richest in lactose (6.6 g per 100 g versus 4.7 g per 100 g in cow [31]), one more reason why we should expect these populations to be highly LP. This peculiar composition turns out to make mare’s milk more amenable to ferment spontaneously [31], and indeed in Central Asia, it is drunk in the form of fermented beverages (belonging to the yeast–lactic fermentation group, in which alcoholic fermentation by yeasts is used in combination with lactic acid fermentation by bacteria). Such beverages (named “kumis” in the former Soviet Central Asian republics, “ajrag” in Mongolia) seem to have already existed since the Scythians [32]. These fermentation and consumption practices are not restricted to mare’s milk, as they also apply to camel’s milk in Kazakhstan [33] and have been found in Bronze Age China with ruminant milk [34]. Whatever the animal source of the milk, it seems that a specificity of Central Asian herders is to never drink raw milk; it is rather heated and added in small amounts to tea, or mostly transformed in dairy products (alcoholic fermented beverages, yogurt, curd, cream, butter) [35].

Even though there is some lactose left in fermented milk, especially in mare’s lactose-rich milk (0.2–4 g per 100 g [2,36–39]), it has been shown that a given amount of lactose is easier to digest in fermented products than in unfermented ones (individuals consuming yogurt and kefir present a reduction of symptoms of 60%–80% compared with unfermented milk [40,41]). This is likely because the bacteria that transform lactose are ingested together with the lactose and survive in the human digestive system, thus reducing the amount of lactose reaching the colon and its side effects [41]. It is further possible that associated changes in the human gut microbiota (notably an increase in *Bifidobacterium* sp. [42]) reinforce the digestibility of lactose and help reduce symptoms. In consequence, the fermentation of dairy products represents a cultural (and possibly colonic) way to adapt to milk consumption, allowing individuals to benefit from all micronutrients from milk, as well as lipids and proteins, without any associated symptoms. Importantly, though, nonpersistent individuals are still not able to derive glucose out of lactose [43,44]. But if individuals are able to get calories and most nutritional benefits (including calcium and vitamin D) from dairy products without genetic adaptation, the question then becomes this: why did some populations strongly select for the genetic ability to digest lactose?

## Why was LP genetically selected for at all?

A first possibility is that not all populations knew how to derive dairy products from milk. This is, however, not very likely, as there is evidence for cheese making in Europe as early as 7,000 BP [45]. Another possibility is that famines and/or drought were less severe in Asia than elsewhere. However, animals are also very sensitive to food shortages, and milk is unavailable during those times. It is also possible that ecological differences among pastoral populations have been associated to differences in the ability to produce, transform, or store dairy products (with, e.g., a possible influence of temperature on fermentation abilities and/or of mobility on conditioning strategies), even though LP populations exist in very diverse ecological settings (from North Europe to East Africa). One common point, though, between North Europeans and Africans LP populations is that they are both traditionally cattle herders, whereas this is not the case in South Europe (where herds consist mostly of goats and sheep) or in Central Asia. This might have led to important differences in the quantity or quality of milk, resulting in a different profile of milk incorporation in the diet. Finally, it is impossible to exclude cultural preferences (based on taste and/or symbolism) resulting in differences in consumption practices.

## Conclusions and future directions

In summary, the −13.910*T allele was first seen in Central Europe 5,950 years ago. Given that most samples around that time do not carry evidence for any steppe ancestry, it is difficult to infer whether it originated in Yamnaya-associated cultures or in European farmers. Regardless, the T allele quickly spread across Eurasia during the late Bronze Age (first appearance at 3,713 BP in Central Asia), which is concomitant with the expansion of Yamnaya-associated cultures. This suggests that steppe populations might have contributed to the spread of the T allele across and outside Europe. This hypothesis is further supported by the fact that the −13.910*T allele is currently found at elevated frequency in Europe and North India (Fig 2), the two places where Yamnaya-associated populations are known to have left some genetic legacy [46]. The T allele then strongly increased in frequency in Europe (reaching 31% in average in the 3,000 BP to present day period) while remaining low in Central Asia (6%), likely reflecting differences in selective pressures between populations.

It is not clear why Central Asian populations seem to have adapted culturally (and possibly by gut microbiota adaptations) while North Europeans and Africans have adapted genetically. Possible explanations include the composition of the herds (with cattle possibly providing larger quantities of milk), ecological differences (e.g., linked to mobility), and/or cultural preferences. Given that very little data exist on how and in what amounts milk is consumed and transformed across traditional herders and farmers worldwide, more comparative anthropological work is thus needed to directly test and assess the validity of different hypotheses. Special attention may be given to the consumption practices and the symptoms in children, which have been mostly overlooked so far. Indeed, differences in lactase expression between nonpersistent and persistent individuals occur as early as 3 years old [47–49], and at that age, the symptoms might be more severe, as they depend on individuals’ weight. Interestingly, it has recently been shown that during early Iron Age in Germany, animal milk was given to young children (1, 1–2, and 0–6 years old, respectively) in ceramic baby bottles [50].

It would also be informative to collect more systematically LP phenotype together with gut microbiome data, to explore whether in some populations, gut bacteria might have actually helped diminish the symptoms associated with drinking milk and thus allowed individuals to adapt to dairy consumption in a nongenetic manner.

Some populations, such as the Tibetans, would also be especially relevant to study in more depth, given that they have relied on pastoralism for a long time [51] and appear to consume mostly fermented products from yak’s milk [52]. So far, only one phenotypic study has been performed (on 30 individuals), showing a LP prevalence of 30% [53], and the genetic basis for LP in these populations is unclear [54]. In parallel, it has recently been shown that there was no significant difference in LP phenotypic frequency between farmers and herders from Iran [55], so ethnographic work could also be done in these populations to see whether their consumption practices match our hypothesis.

In conclusion, the widespread idea that LP has been genetically selected for in all populations whose ancestors had access to milk because of calorific content is not valid, and it seems that cultural adaptation (by the external use of bacteria to digest lactose during fermentation) and possibly colonic adaptation (by seeding the gut with beneficial bacteria) allowed some populations to develop pastoral dairying cultures without having to genetically adapt to lactose digestion.

## Methods

### Genotyping modern DNA samples in Central Asia

Given that the −13.910:C>T mutation has been shown to be strongly and significantly correlated with the LP phenotype throughout Eurasia (in a dominant manner, genotype-phenotype correlation of 0.973 [56]), including in Central Asia (all LP individuals were found to carry the −13.910*T allele [16]), we could infer individuals’ phenotype based on their genotype at this locus. We used various molecular methods: 183 individuals from 2 populations were genotyped by PCR [16], 441 individuals from 17 populations were genotyped by RFLP [11], 356 individuals from 12 populations were genotyped on an Illumina Omni1 genotyping array, and 56 individuals from 2 populations were genotyped on an Illumina Omni2.5 genotyping array [57].

### Inferring the −13.910*T allele frequency from ancient DNA

We used a publicly available compilation of multiple ancient DNA studies from David Reich Lab’s website [58], which we merged with 3 additional papers [28,46,59], summing to 3,006 individuals, of which 1,662 had at least one read overlapping the −13.910 position. Retaining only individuals ranging from 10,000 BP to present day from Eurasia (where the −13.910*T allele is the only one associated with LP), we obtained data for 1,434 individuals.

Given that ancient DNA sequences mostly have a very low coverage and a non-negligible genotyping error rate, diploid genotype calls are difficult to obtain for most individuals. To overcome this issue and obtain reliable allele frequency estimations, the authors of these papers have haploidized individuals by randomly picking one read [58]. However, this approach results in nonreliable individual calls, as heterozygous C/T individuals will be considered negative half the time in average. Thus, in parallel, we also analyzed the raw BAM files (see next section).

For the comparison between Europe and Central Asia, we used a cutoff at a longitude of 45° (corresponding to the Volga river) to discriminate between the 2 regions. We excluded individuals from Turkey, Israel, Lebanon, Jordan, Iran, and Pakistan for these analyses. The difference between the 2 areas was tested with R using a two-sample test for equality of proportions with continuity correction.

### Inferring individual diploid calls from ancient DNA

To complete the previous approach and obtain reliable individual genotype calls, we downloaded available aligned BAM files for the papers included in our previous dataset and retained individuals carrying at least 3 reads with a C or 3 reads with a T, corresponding to 874 individuals. We considered individuals as LP if they had at least 3 reads carrying a T (and if this corresponded to more than 20% of the total number of reads). This alternative approach results in losing an important number of individuals but allows us to confidently identify which individuals are true positives. We provide the map corresponding to this approach in S2 Fig.

## Supporting information

S1 Fig Temporal dynamic of the −13.910*T allele frequency in Eurasia with more temporal resolution. The color scale represents the extrapolated T allele frequency. Light/dark blue points represent individuals for which one randomly chosen read carries the C/T allele.
(TIF)

S2 Fig Temporal dynamic of the phenotypic frequency of LP in Eurasia. The color scale represents the extrapolated frequency of LP. For the 3 ancient maps, light/dark blue points represent lactase nonpersistent and persistent individuals, respectively. For the modern map, see the legend of Fig 1.
(TIF)

S1 Table Contextual information on the ancient samples used for the spatiotemporal analysis of the −13.910*T allele frequency (consisting of 1,434 individuals, of which 874 had enough coverage to call genotypes and thus were used to infer the frequency of LP). The data are taken from David Reich Lab’s website [58], to which we added information for 3 additional newer publications [28,46,59]. The number in the “rs4988235” column corresponds to the number of reference allele at the −13.910 position (thus 0 means the alternative T allele, and 2 means the reference C allele). This is the information used in Fig 2 and S1 Fig. The last column (“LP status”) gives the phenotypic status of each individual based on its genotype at all reads (see Methods). This is the information used in S2 Fig.
(XLSX)

## Abbreviations

BP: before present
GLAD: Global Lactase persistence Association Database
LBK: Linearbandkeramik
LP: lactase persistence
