# Supplementary figures and images for: Why and when was lactase persistence selected for? Insights from Central Asian herders and ancient DNA
Source: PLoS Biol. 2020 Jun 8;18(6):e3000742. doi: 10.1371/journal.pbio.3000742 (PMC7302802; doi:10.1371/journal.pbio.3000742)

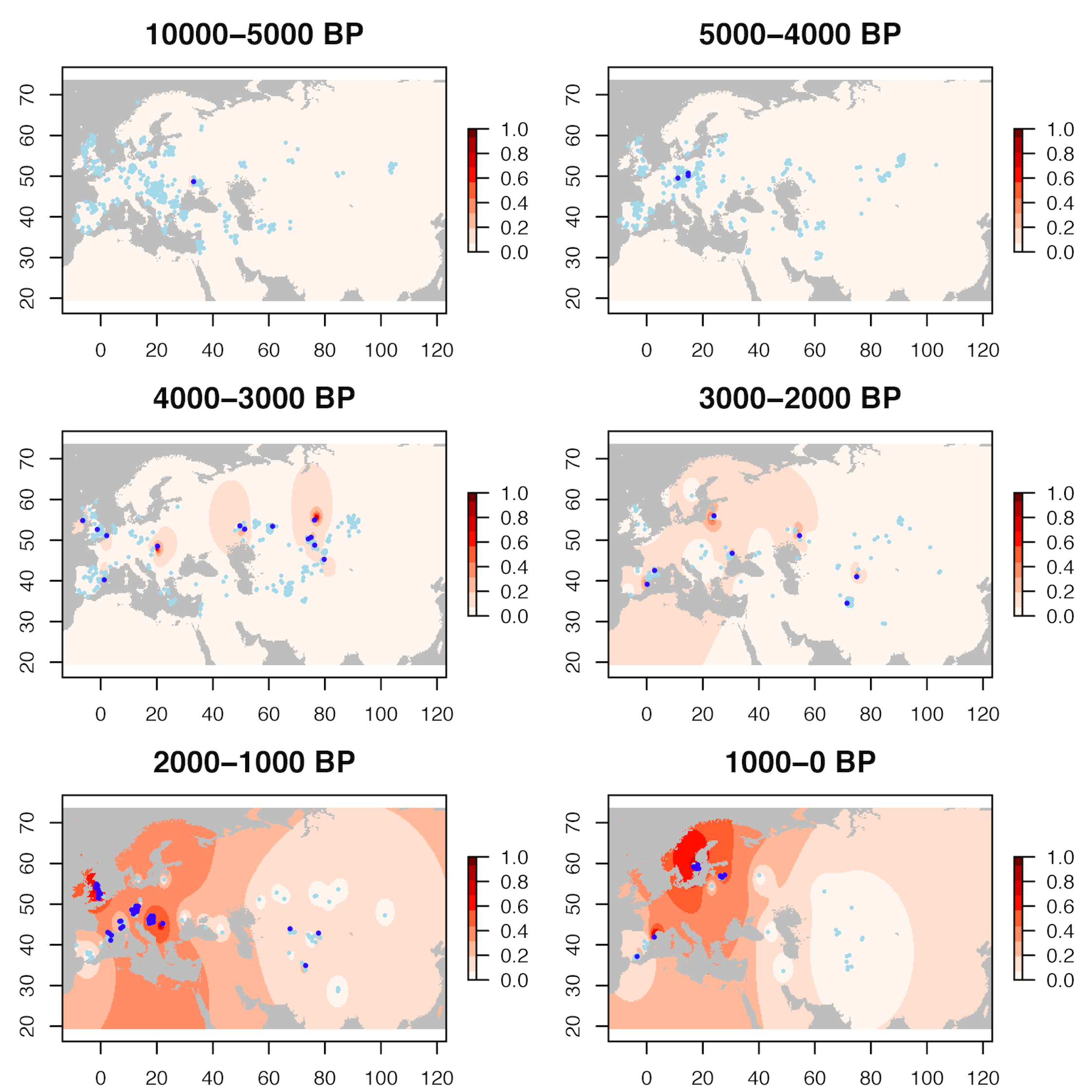

Supplement: S1 Fig — The color scale represents the extrapolated T allele frequency. Light/dark blue points represent individuals for which one randomly chosen read carries the C/T allele. (TIF) [file pbio.3000742.s001.tif]

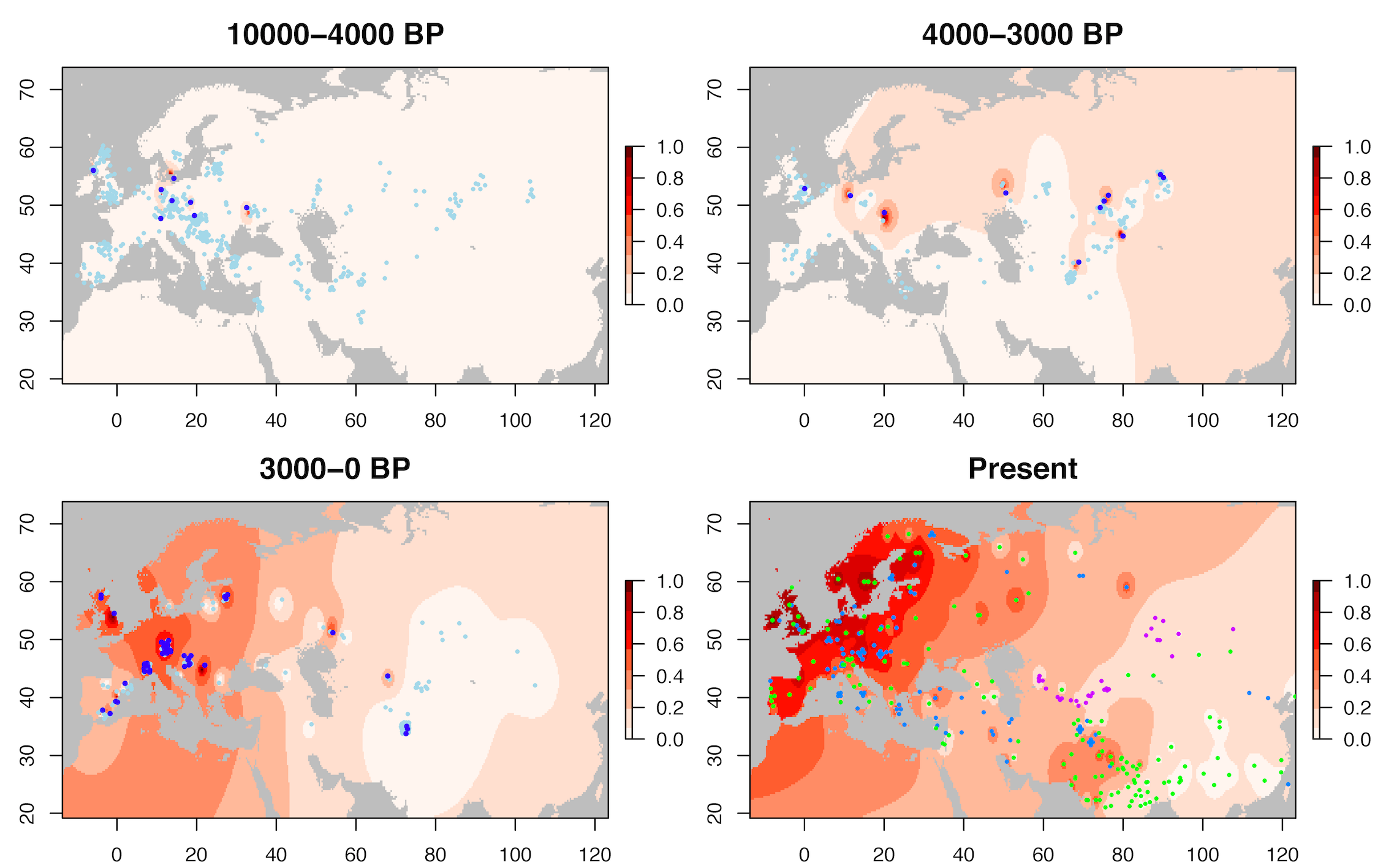

Supplement: S2 Fig — The color scale represents the extrapolated frequency of LP. For the 3 ancient maps, light/dark blue points represent lactase nonpersistent and persistent individuals, respectively. For the modern map, see the legend of Fig 1. (TIF) [file pbio.3000742.s002.tif]
